# Exposures to Particulate Matter from the Eruptions of the Puyehue Volcano and Birth Outcomes in Montevideo, Uruguay

**DOI:** 10.1289/EHP235

**Published:** 2016-05-06

**Authors:** Ana Ines Balsa, Marcelo Caffera, Juanita Bloomfield

**Affiliations:** 1 Department of Economics, University of Montevideo, Montevideo, Uruguay; 2 Tinbergen Institute, University of Amsterdam, Amsterdam, the Netherlands

## Abstract

**Background:**

The ashes and dust resulting from the 2011 eruptions of the Puyehue volcano in Chile more than doubled monthly averages of PM10 concentrations in Montevideo, Uruguay. Few studies have taken advantage of natural experiments to assess the relationship between ambient air pollutant concentrations and birth outcomes.

**Objectives:**

In this study we explored the effect of particulate matter with diameter of ≤ 10 μm (PM10) on perinatal outcomes in Uruguay, a middle-income country in South America with levels of PM10 that in general do not exceed the recommended thresholds. The analyzed outcomes are preterm birth, term birth weight, and term low birth weight.

**Methods:**

We took advantage of the sharp variation in PM10 concentrations due to the Puyehue eruptions to estimate the associations between mother’s exposure to PM10 in each trimester of pregnancy and perinatal outcomes. We use birth registries for 2010–2013 and control for covariates, including maternal and pregnancy characteristics, weather, co-pollutants, and calendar quarter and hospital indicators.

**Results:**

A 10-μg/m3 increase in exposure to PM10 during the third trimester was associated with a higher likelihood of a preterm birth [odds ratio (OR) = 1.10; 95% CI: 1.03, 1.19]. The association was robust to different model specifications, and increased with categorical exposure levels (OR for third-trimester PM10 ≥ 70 vs. < 30 μg/m3 = 5.24; 95% CI: 3.40, 8.08). Exposures were not consistently associated with birth weight or low birth weight among term births, though second-trimester exposures were associated with higher birth weight, contrary to expectations.

**Conclusions:**

Taking advantage of a natural experiment, we found evidence that exposure to high levels of PM10 during the third trimester of pregnancy may have increased preterm births among women in Montevideo, Uruguay.

**Citation:**

Balsa AI, Caffera M, Bloomfield J. 2016. Exposures to particulate matter from the eruptions of the Puyehue Volcano and birth outcomes in Montevideo, Uruguay. Environ Health Perspect 124:1816–1822; http://dx.doi.org/10.1289/EHP235

## Introduction

The Puyehue Cordon Caulle volcanic complex in Chile experienced a series of eruptions between June and November of 2011. Following these events, clouds of dust covered the city of Montevideo, Uruguay. During June and July, daily concentrations of particulate matter ≤ 10 μm (PM_10_) in Montevideo exceeded the World Health Organization 24-hr mean guideline of 50 μg/m^3^ (WHO 2006) in 60% of the days, and were > 100 μg/m^3^ in 30% of the days. The eruption in November caused a similar increase in PM_10_ concentrations. In this study we took advantage of this natural experiment to analyze the association between exposure to PM_10_ and preterm birth (PTB), term birth weight (BW), and term low birth weight (LBW).

LBW and PTB are commonly used as proxies for infant health and are markers for poor health during the life course (Black et al. 2007; Boardman et al. 2002; McCormick 1985; Oreopoulos et al. 2008; Petrou et al. 2001). LBW has been associated with higher morbidity and lifetime health costs, as well as lower academic achievement, lower income, and early mortality (Almond et al. 2005; Behrman and Rosenzweig 2004; Currie 2009; Figlio et al. 2014; Rosenzweig and Zhang 2013; Royer 2009). Moreover, there is evidence of a strong intergenerational correlation in the BW of mothers and children (Case et al. 2004; Currie 2009; Currie and Madrian 1999; Currie and Moretti 2005; Grossman 2000).

Although many studies have analyzed the association between ambient air pollutant concentrations and birth outcomes (Currie et al. 2009; Dadvand et al. 2013; Parker et al. 2011; Šrám et al. 2005; Stieb et al. 2012; Woodruff et al. 2009), only a few (Huang et al. 2015; Parker et al. 2008; Rich et al. 2015) have approached the issue by using a natural experiment. Parker et al. (2008) compared pregnancies exposed to the Utah Valley Steel Mill (Utah, USA) closure that occurred between mid-1986 and mid-1987 to pregnancies in pre-and postclosure periods. They found that mothers who were pregnant around the time of the closure of the mill were less likely to deliver prematurely than mothers who were pregnant before or after the mill closure. Similarly, Rich et al. (2015) compared pregnancies exposed to the air pollution declines during the 2008 Beijing Olympics to pregnancies before and after the Olympic games. Their results showed that exposure to lower levels of air pollution late in pregnancy were associated with higher BW. Huang et al. (2015) also took advantage of the reduction in air pollution during the 2008 Olympics in Beijing, but found no relationship between PM_10_ concentration and term BW or PTB. Other studies using natural experiments to assess the effects of pollution on perinatal outcomes are those by Chay and Greenstone (2003a, 2003b) and Currie and Walker (2011). However, none of them focused on the effects of mother’s exposure to PM_10_ on birth outcomes.

Our study contributes to this literature by estimating associations between birth outcomes and variation in pollution resulting from a volcano eruption, a natural and completely unexpected event. It is also one of a few studies to report the association between PM_10_ and birth outcomes in Latin America.

## Methods

### Data

***Pregnancy and delivery data.*** We analyzed live births that took place in Montevideo during 2010–2013 and that were registered in the Perinatal Information System (Mainero 2010). The Perinatal Information System is a mandatory electronic registry of perinatal histories covering about 98% of all pregnancies in the country. Because the data were anonymous, approval from a review board was not required.

The outcomes of interest were PTB, BW for full-term pregnancies, and LBW for full-term pregnancies. We defined a PTB as a delivery occurring before the 37th week of gestation. BW was measured in grams. LBW was a binary variable that took the value of 1 if the BW was ≤ 2,500 g, and 0 otherwise.

We addressed potential confounding by adjusting for several maternal characteristics that may contribute to maternal and pregnancy heterogeneity: mother’s age (< 20, 20–34, 35–39, ≥ 40 years), education level (less than middle school, middle school completed, or high school completed), marital status (common law, married, single, other), eclampsia or hypertension during the pregnancy (separate variables based on birth record information; yes/no), maternal smoking during pregnancy (yes/no), body mass index (BMI) before pregnancy (based on mother’s recall at first visit; underweight, BMI < 18.5; normal, 18.5 ≤ BMI < 25; overweight, 25 ≤ BMI < 30; obese, BMI ≥ 30), parity (continuous), onset of prenatal care (gestational week, continuous), and the child’s sex.

Our analysis also accounted for health-care heterogeneity by adjusting for 22 binary indicators for the 23 hospitals in the city. Of these, 10 were public, covering the poorest fraction of the population (40% of all deliveries), and the rest were private hospitals associated with health maintenance organizations that provide services to privately insured individuals or to workers in the formal labor market and their dependents through the national social insurance (National Integrated Health System).

We dropped multiple births and births with BW < 300 g or > 8,000 g. To avoid the problem of fixed cohort bias raised by Strand et al. (2011), we restricted our sample to pregnancies conceived between 1 June 2009 and 1 April 2013. We were not able to distinguish multiple pregnancies to the same mother; therefore, eligible births may include more than one pregnancy in the same woman.

***Air quality data.*** The air quality data came from the Environmental Control and Quality Evaluation Service of the Municipal Government of Montevideo. This office is in charge of the city’s air quality monitoring network. In 2009 the network incorporated an automatic station in the area of Colón, north of Montevideo, measuring air quality [PM_10_, sulfur dioxide (SO_2_), carbon monoxide (CO), and nitrogen dioxide (NO_2_)] on an hourly basis. This was the only automatic monitoring station in Montevideo operating throughout the full period of analysis (2009–2013).

Although three other manual stations in the city collected data on PM_10_ between 2009 and 2013, we chose not to work with these other sources for two reasons: First, samples in the manual stations were obtained every 6 days and were more likely to miss extreme episodes, such as days with abnormal levels of ashes (IMM 2016). Second, our analysis of data from these manual stations (data not shown; available upon request), shows that most of the variation in PM_10_ levels occurred over time for the full city, rather than between city areas. Unreported analysis of variance for the period 2009–2013 shows that the variation in PM_10_ resulting from the volcanic eruption was almost three times higher than the intraneighborhood variation in air quality in Montevideo.

Our variable of interest is ambient air 24-hr mean concentration of PM_10_, averaged at the trimester-of-pregnancy level. Specifically, we calculated the week of initiation of the pregnancy by subtracting the gestational age at birth, as assessed by the obstetrician at delivery, from the date of birth, and then adding 2 weeks to account for the difference between gestational age (which is based on the last menstrual period) and the date of conception. For each pregnancy, we matched each week with the corresponding average PM_10_ for that week, and then computed the average exposure to PM_10_ in the first, second, and third trimesters of pregnancy. The first trimester runs from conception to week 13, and the second trimester from week 14 to week 27. Exposure to PM_10_ during the third trimester depends on the term of gestation. When analyzing the probability of a PTB, we computed the third-trimester values by averaging PM_10_ levels between gestation week 28 and gestation week 36 if the pregnancy reached full term, or between gestation week 28 and the week of delivery if the birth occurred before week 37. When analyzing outcomes for full-term births (BW and LBW), we considered the average exposure to PM_10_ for the full third trimester.

***Weather data and other controls.*** We obtained 24-hr mean averages of temperature (degrees Celsius), air pressure (hectopascals; hPa), windspeed (meters per second), and humidity (percent) from three weather-monitoring stations of the National Institute of Meteorology located in the East, North, and West of Montevideo (Carrasco, Prado, and Melilla). We also obtained from these same stations the accumulated level of precipitation over 24 hr, measured in millimeters per square meter. For each weather variable we averaged out these measures across the three stations and constructed trimester-of-pregnancy–specific averages following the same procedure as with PM_10_.

### Statistical Analysis

***Estimation procedure.*** We estimated the associations between a pregnant mother’s average exposure to PM_10_ in each trimester of her pregnancy and three perinatal outcomes: PTB, BW, and LBW. We considered all births when analyzing PTB, but only full-term births when the outcomes were BW and LBW. By restricting the analysis of these two outcomes to non-premature pregnancies, we sought to isolate potential associations between PM_10_ and intrauterine growth retardation. Our identification strategy relied on the exogenous variation of PM_10_ concentration in Montevideo that resulted from the Puyehue ashes.

We estimated associations between exposure to PM_10_ during the pregnancy and BW with ordinary least squares, and used logistic models for the dichotomous outcomes (PTB, LBW). We set the statistical significance level (α) at 0.05. For PM_10_ as a continuous variable, our model took the form:

*Y* = *f*(α + β_1_ *PM10*_*T1* + β_2_ *PM10*_*T2* + β_3_ *PM10*_*T3* + δ***X*** + γ***Z*** + λ**μ** + φ***Q_t_***) [1]

where *Y* is PTB, LBW, or BW; and *f*(.) is a linear function when the outcome is BW and a logistic function when analyzing PTB or LBW. We included all births when analyzing PTB, but only full-term births when the outcome was BW or LBW. *PM10_T1*, *PM10_T2*, and *PM10_T3* represent average exposures to PM_10_ during the first, second, and third trimester, respectively. The vector ***X*** represents maternal covariates, ***Z*** represents the five weather variables in each trimester (15 variables total), and **μ** represents the 22 indicator variables for the 23 prenatal care centers in the study area. In addition, we adjusted for ***Q_t_***, a vector of 15 indicator variables for the possible 16 combinations of calendar quarter and year of conception in the sample (the earliest date of conception in our data was June 2009 and the latest March 2013). The latter captures underlying trends and seasonality in perinatal outcomes (Currie and Schwandt 2013).

In a second specification, for each trimester *t* we modeled three dichotomous indicator variables for PM_10_ categorized as 30–49 μg/m^3^ (*PM30_49t*), 50–69 μg/m^3^ (*PM50_69t*), and ≥ 70 μg/m^3^ (*PM70t*), with PM_10_ < 30 μg/m^3^ serving as the reference exposure category. We refer to the estimations resulting from this specification as the categorical PM_10_ analysis.

We conducted, in addition, several robustness tests. Because the consistency of our estimates relies on the exogeneity of PM_10_ variation over time, we ran two additional regressions that controlled for potential confounders. The first regression added a set of adjustors to the core categorical regression that were potentially associated with the concentration levels of PM_10_. These included the level of activity of two thermal plants, measured in megawatt-hours (MWh), and obtained from the Electric Market Administration Office, and the production volume of the oil refinery, a production index with base 2006 = 100 constructed by the National Institute of Statistics. For both measures, we computed trimester-of-pregnancy–specific averages on the basis of the available monthly measures.

The second regression added controls for NO_2_ (μg/m^3^), SO_2_ (μg/m^3^), and CO (μg/m^3^) to the core categorical analysis. These co-pollutants were averaged at the trimester of pregnancy level in the same way as the PM_10_ and weather variables. We ran regressions controlling first for one co-pollutant at a time, and then adding the three in the same estimation. We did not have complete data on CO, SO_2_, and NO_2_. Twenty-four-hour data were missing on 0.17% of the days for CO, on 10% of the days for NO_2_, and on 6% of the days for SO_2_. Because there were no large periods without data, we disregarded these days with missing values when constructing averages at the trimester of pregnancy level. By doing so, we had no missing data on trimester averages of these copollutants for full-term births. However, for a few number of deliveries with low gestational age, we had some missing values for the third trimester. In particular, CO was missing for 31 observations, NO_2_ was missing for 51 observations, and SO_2_ was missing for 5 observations. This explains why the number of observations in the analyses of PTB varies when adjusting for different copollutants, whereas the number of observations is the same, regardless of the copollutant, in the analyses of BW and LBW.

A third sensitivity check addressed the issue of missing values on eclampsia, hypertension, parity, and smoking. In the core analysis we imputed the corresponding mean value to the observations with missing data on one or more of these variables, and added a dichotomous indicator equal to 1 when the observation had a missing value on the variable, and 0 otherwise. The only variables with missing data were eclampsia, hypertension, parity, and smoking. For categorical variables, we imputed the average proportion of women with the characteristic. We used one separate dichotomous indicator of missing data for each of the four covariates. The purpose of these indicators was to absorb any differential variation on observations with missing data, without having to rely on the artificially imputed value (which was constant across all observations with a missing value). The estimates could be biased if women with missing observations on these variables were different from other women, and the fraction of these women was changing over time. We explored this issue by running the analysis only for observations without missing values on eclampsia, hypertension, parity, and smoking.

Fourth, to assess the sensitivity to the reference group used for comparison, we first restricted our estimation to pregnancies with a date of delivery before or during the volcano eruptions, and then to pregnancies with a conception date after the first eruption (restricting in this case the sample to pregnancies exposed to the ashes and pregnancies post-eruption). We also estimated associations with PM_10_ (categorical and continuous) for pregnancies that were not exposed to the volcano eruptions (deliveries before 8 June 2011 and pregnancies with a date of conception after 30 December 2012). During these periods, only 156 pregnancies had exposures ≥ 50 μg/m^3^ in any trimester.

Fifth, the inclusion of several variables in the same regression measuring pollution and weather by trimester raises the challenge of multicollinearity and its potential consequences on the precision of standard errors. To test for this possibility, we followed Bell et al. (2007) and used residuals of trimester averages regressed on the average of a reference trimester. For simplicity, we conducted this robustness check only on the specification using a single average by trimester. For example, we selected the first trimester as the reference trimester and then regressed PM_10_ (and weather) averages for the second and third trimester on the first trimester. We reran the estimations using residuals of the instrumental regressions for the second and third trimesters, as well as the average for the reference category. We repeated this exercise alternating the reference trimester.

## Results

Table 1 provides descriptive statistics for the main variables in the analysis by time period (before, during, and after the Puyehue eruption). The proportion of preterm births in the full period is 8%; 7% occurred between weeks 32 and 36 of gestation, and 1% took place between gestational weeks 28 and 31. Among full-term births, 2.7% were low weight. The average weight for a full-term baby was 3,354 g. Almost 70% of women belonged to the 20–34 years age range, 32% were high school graduates, and 37% had not completed middle school. The majority of mothers (54%) lived under common law, 27% were married, and 18% were single. Almost one of four women reported smoking during the pregnancy (missing data for 0.6% of the sample). The majority of women initiated prenatal care during week 12 of gestation. Overall, our data had 79,328 observations on pregnancies, 26,266 of which showed exposure to high levels of particulate matter in June, July, or November of 2011 due to the ashes from the Puyehue eruption. We observed 24,906 pregnancies with delivery dates before the volcano eruption and 28,156 pregnancies with conception dates after the eruption.

**Table 1:** Descriptive statistics, by exposure to Puyehue ashes*^a^* (*n* = 79,328).

Characteristic^*b*^	Before eruption (*n* = 24,906)	During eruption (*n* = 26,266)	After eruption (*n* = 28,156)
Individual-level variables
Pregnancy outcomes
Preterm birth (< 37 weeks)	2,191 (8.8)	2,045 (7.8)	2,172 (7.7)
Birth weight (g; full-term births only)	3,335 ± 456	3,361 ± 451	3,364 ± 452
Low birth weight (full-term births only)	693 (3.1)	586 (2.4)	660 (2.5)
Maternal age (years)
< 20	4,098 (16.5)	4,260 (16.2)	4,845 (17.2)
20 ≤ age ≤ 34	17,239 (69.2)	17,864 (68.0)	18,932 (67.2)
35 ≤ age ≤ 39	2,932 (11.8)	3,407 (13.0)	3,587 (12.7)
> 40	637 (2.6)	735 (2.8)	792 (2.8)
Maternal education
Less than middle school	9,824 (39.4)	9,696 (36.9)	10,243 (36.4)
Middle school < education < high school	7,481 (30.0)	7,901 (30.1)	8,453 (30)
Completed high school	7,601 (30.5)	8,669 (33)	9,460 (33.6)
Maternal marital status
Common law	13,486 (54.1)	14,263 (54.3)	15,712 (55.8)
Married	6,750 (27.1)	7,112 (27.1)	7,168 (25.5)
Single	4,437 (17.8)	4,669 (17.8)	5,061 (18.0)
Other marital status	233 (0.9)	222 (0.8)	215 (0.8)
Pregnancy conditions
Eclampsia	47 (0.2)	35 (0.1)	33 (0.1)
Eclampsia missing	3,097 (12.4)	2,154 (8.2)	1,405 (5.0)
Hypertension	572 (2.3)	561 (2.1)	661 (2.3)
Hypertension missing	3,048 (12.2)	2,127 (8.1)	1,382 (4.9)
Mother underweight^*c*^	1,597 (6.4)	1,562 (5.9)	1,683 (6.0)
Normal BMI^*c*^	17,215 (69.1)	17,886 (68.1)	18,659 (66.3)
Mother overweight^*c*^	4,171 (16.7)	4,564 (17.4)	5,221 (18.5)
Mother obese^*c*^	1,923 (7.7)	2,254 (8.6)	2,593 (9.2)
Mother smokes	6,179 (24.8)	6,062 (23.1)	6,411 (22.8)
Smoking status missing	288 (1.2)	97 (0.4)	67 (0.2)
Parity	1.15 ± 1.33	1.11 ± 1.33	1.08 ± 1.28
Parity missing	3,344 (13.4)	2,962 (11.3)	3,470 (12.3)
Newborn sex: male	12,582 (50.5)	13,401 (51)	14,432 (51.3)
Week of initiation of prenatal care^*d*^	12.93 ± 7.55	11.90 ± 7.09	11.40 ± 6.71
Pollution variables
First-trimester PM_10_ (μg/m^3^)	20.4 ± 4.2	45.5 ± 17.4	23.7 ± 8.7
< 30 μg/m^3^	24,544 (98.5)	6,039 (23.0)	20,867 (74.1)
30–49 μg/m^3^	362 (1.5)	7,937 (30.2)	7,289 (25.9)
50–69 μg/m^3^	0 (0)	9,768 (37.2)	0 (0)
≥ 70 μg/m^3^	0 (0)	2,522 (9.6)	0 (0)
CO 1st trimester (μg/m^3^)	0.49 ± 0.03	1.14 ± 0.64	0.62 ± 0.12
CO missing in any trimester	30 (0.12)	1 (0.00)	0 (0.00)
NO_2_ 1st trimester (μg/m^3^)	30.09 ± 9.84	24.48 ± 8.02	24.65 ± 8.58
NO_2_ missing in any trimester	6 (0.02)	45 (0.17)	0 (0.00)
SO_2_ 1st trimester (μg/m^3^)	17.93 ± 6.30	11.50 ± 5.54	6.77 ± 3.23
SO_2_ missing in any trimester	5 (0.02)	0 (0.00)	0 (0.00)
Weather variables
Precipitation 1st trimester (mm)^*e*^	3.68 ± 0.91	2.36 ± 0.88	3.58 ± 1.07
Temperature 1st trimester (°C)^*f*^	15.89 ± 3.96	18.11 ± 4.25	17.78 ± 4.08
Windspeed 1st trimester (m/sec)^*f*^	14.26 ± 0.84	14.60 ± 1.50	13.43 ± 1.59
Humidity 1st trimester (%)^*f*^	74.00 ± 2.37	69.81 ± 4.03	73.51 ± 4.88
Atmospheric pressure 1st trimester (hPa)^*f*^	1015.87 ± 2.89	1014.93 ± 2.45	1014.89 ± 2.36
Correlations between pollutants^*g*^
PM_10_ and SO_2_ 1st trimester	0.73 (*p* = 0.00)	–0.36 (*p* = 0.00)	0.39 (*p* = 0.00)
PM_10_ and NO_2_ 1st trimester	–0.36 (*p* = 0.00)	–0.13 (*p* = 0.00)	–0.27 (*p* = 0.00)
PM_10_ and CO 1st trimester	–0.34 (*p* = 0.00)	–0.20 (*p* = 0.00)	0.13 (*p* = 0.00)
Values are *n* (%) or mean ± SD, unless otherwise indicated. ^***a***^Pregnancies classified as “before,” “during,” and “after” eruption were conceived June 2009–September 2010, October 2010–November 2011, and December 2011–March 2013, respectively. ^***b***^In addition to the above-mentioned variables, our analysis adjusts for 22 binary indicators for the 23 hospitals in the country. ^***c***^Underweight: BMI < 18.5; normal: 18.5 ≤ BMI < 25; overweight: 25 ≤ BMI < 30; obese: BMI ≥ 30. ^***d***^Gestational week, continuous. ^***e***^Trimester of pregnancy means of 24 hr accumulated precipitation. ^***f***^Trimester of pregnancy means of 24 hr averages. ^***g***^Correlation coefficients.			

**Figure 1:**
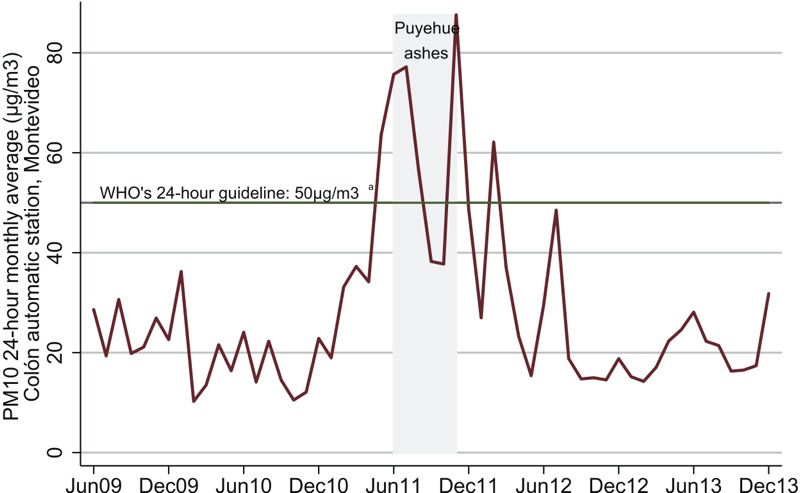
PM_10_ monthly averages in Montevideo. ***^a^*** WHO (2006).

When averaged at the trimester level, the standard deviation of PM_10_ over time (i.e., within stations) was 14, whereas the standard deviation between stations was 5.5. Figure 1 shows monthly averages of PM_10_ in Montevideo and highlights the dates when the volcanic ashes from the Puyehue arrived in the city. The mean level of PM_10_ during the first trimester was 21.2 ± 5.9 μg/m^3^ for pregnancies not exposed to the Puyehue ashes and 46 ± 17.4 μg/m^3^ for pregnancies exposed to the ashes (Table 1). Averages for the second and third trimesters (data not shown) were similar to the first-trimester averages shown in Table 1. Table 1 shows also that almost half of the pregnancies during the Puyehue period were exposed to trimester-average levels of PM_10_ > 50 μg/m^3^. On the contrary, none of the pregnancies before or after the eruptions were exposed to trimester-average levels of PM_10_ this high.

Table 1 shows also descriptive statistics for other pollutants, including CO, NO_2_, and SO_2_. As in the case of PM_10_, average levels of CO increased during the volcano period and then returned to prior levels. There was no evidence of increases in the levels of NO_2_ and SO_2_. Furthermore, we did not find correlations of magnitude between PM_10_ and other pollutants. Correlation coefficients change signs in the different periods of analysis, suggesting a noisy relationship between the pollutants.

### Continuous PM_10_ Analysis

We found a positive association between average PM_10_ exposure during the third trimester and PTB (Table 2). A 10-μg/m^3^ increase in average PM_10_ during the third trimester was associated with a 10% increase in the odds of PTB [odds ratio (OR) = 1.10; 95% confidence interval (CI): 1.03, 1.19]. On the other hand, we did not find evidence of adverse associations between PM_10_ and term BW or term LBW. We did find, however, a positive small association between PM_10_ concentrations during the second trimester and BW. A 10-μg/m^3^ increase in PM_10_ during the second trimester was associated with a 13-g higher birth weight among term births (95% CI: 4.07, 22.13).

**Table 2:** Preterm birth (all pregnancies, *n* = 79,328), and low birth weight (< 2,500 g) and birth weight (g) among term births (*n* = 72,920) in association with a 10-μg/m^3^ increase in average PM_10_ during each trimester.

Exposure	Preterm birth [OR (95% CI)]	Birth weight (g) [coefficient (95% CI)]	LBW [OR (95% CI)]
1st trimester	0.97 (0.91, 1.06)	–3.03 (–11.27, 5.22)	1.04 (0.93, 1.15)
2nd trimester	0.96 (0.89, 1.05)	13.10 (4.07, 22.13)**	1.01 (0.89, 1.15)
3rd trimester	1.10 (1.03, 1.19)**	–5.78 (–14.90, 3.35)	0.94 (0.81, 1.08)
Model fit
Akaike Information Criterion (AIC)	43,845	1,094,481	17,301
Bayesian Information Criterion (BIC)	44,550	1,095,180	18,000
Adjusted for maternal age, education, marital status, pregnancy conditions, maternal smoking status, and onset of prenatal care; temperature, rain, humidity, air pressure, and windspeed intensity in each trimester of pregnancy; indicators for calendar quarter of gestation; and indicators for prenatal care center. Third-trimester values for PM_10_ and weather variables are averaged across gestation weeks 28 and 36 (or an earlier week if the pregnancy did not reach full term) when the outcome is PTB. In the case of BW or LBW, third-trimester averages consider the full length of the trimester until birth. **p < 0.01.			

### Categorical PM_10_ Analysis

PM_10_ during the third trimester was significantly associated with PTB, with OR = 1.42 (95% CI: 1.07, 1.89) for 50–69 μg/m^3^ and OR = 5.24 (95% CI: 3.40, 8.08) for ≥ 70 μg/m^3^ compared with < 30 μg/m^3^ (Table 3). We also found significant associations between PM_10_ and PTB in the case of the first and second trimesters, but in these cases the ORs were < 1: OR = 0.69 (95% CI: 0.47, 1.02) for ≥ 70 μg/m^3^ in the first trimester, OR = 0.79 (95% CI: 0.64, 0.97) for 30–49 μg/m^3^ in the second trimester, and OR = 0.76 (95% CI: 0.59, 0.99) for 50–69 μg/m^3^ in the second trimester, compared with < 30 μg/m^3^ (Table 3).

**Table 3:** Preterm birth (all pregnancies, *n* = 79,328) and low birth weight (< 2,500 g) and birth weight (g) among term births (*n* = 72,920) in association with PM_10_ during each trimester.

Exposure	Preterm birth [OR (95% CI)]	Birth weight (g) [coefficient (95% CI)]	LBW [OR (95% CI)]
First trimester
< 30 μg/m^3^	Reference	Reference	Reference
30–49 μg/m^3^	0.92 (0.780, 1.08)	–10.03 (–26.78, 6.73)	1.05 (0.83, 1.33)
50–69 μg/m^3^	0.85 (0.66, 1.08)	–3.76 (–28.16, 20.64)	0.98 (0.68, 1.40)
≥ 70 μg/m^3^	0.690 (0.47, 1.02)^#^	–24.26 (–70.81, 22.29)	1.23 (0.64, 2.37)
Second trimester
< 30 μg/m^3^	Reference	Reference	Reference
30–49 μg/m^3^	0.79 (0.64, 0.97)*	16.83 (–2.49, 36.15)^#^	0.98 (0.74, 1.29)
50–69 μg/m^3^	0.76 (0.59, 0.99)*	31.28 (1.89, 60.67)*	1.14 (0.74, 1.74)
≥ 70 μg/m^3^	0.86 (0.58, 1.28)	103.98 (60.89, 147.06)**	0.77 (0.41, 1.45)
Third trimester
< 30 μg/m^3^	Reference	Reference	Reference
30–49 μg/m^3^	1.00 (0.85, 1.17)	–7.96 (–26.02, 10.10)	0.83 (0.63, 1.08)
50–69 μg/m^3^	1.42 (1.07, 1.89)*	57.55 (29.24, 85.86)**	0.52 (0.35, 0.78)**
≥ 70 μg/m^3^	5.24 (3.40, 8.08)**	17.89 (–24.55, 60.34)	0.78 (0.43, 1.42)
Model fit
Akaike Information Criterion (AIC)	43,741	1,094,438	17,298
Bayesian Information Criterion (BIC)	44,502	1,095,192	18,052
Adjusted for maternal age, education, marital status, pregnancy conditions, maternal smoking status, and onset of prenatal care; temperature, rain, humidity, air pressure, and windspeed intensity in each trimester of pregnancy; indicators for calendar quarter of gestation; and indicators for prenatal care center. Third trimester values for PM_10_ and weather variables are averaged across gestation weeks 28 and 36 (or an earlier week if the pregnancy did not reach full term) when the outcome is PTB. In the case of BW or LBW, 3rd trimester averages consider the full length of the trimester until birth. **p < 0.01. *p < 0.05. ^#^*p* < 0.10.			

The two last rows in Tables 2 and 3 compare the goodness of fit of each model under the Akaike Information Criterion (AIC) and the Bayesian Information Criterion (BIC). Both criteria suggest that the categorical model fits the data better when the outcome is PTB. The choice is less clear when analyzing term BW and term LBW: The Akaike information criterion indicated that the categorical model fits the data better, whereas the linear model is better according to the BIC criterion.

### Sensitivity and Robustness

Table S1 depicts the results of the categorical analysis adding controls for the level of activity of two thermal power stations and an oil refinery in Montevideo, which could potentially be correlated with PM_10_. Results were robust to this expanded set of controls (Table S1).

Table S2 shows the results of categorical analysis when adding controls for NO_2_, SO_2_, and CO averages in each trimester of pregnancy to the set of core controls. The addition of these variables resulted in fewer observations in the analysis of preterm births, due to missing data on third-trimester averages of these variables for some women delivering a few days into the third trimester. The association of PTB with third-trimester exposure was stronger after adjustment for all three air pollution variables, with OR = 1.67 (95% CI: 1.19, 2.35) for 50–69 μg/m^3^ (vs. 1.42; 95% CI: 1.07, 1.89 for the default model) and OR = 16.35 (95% CI: 9.26, 28.88) for ≥ 70 μg/m^3^ (vs. 5.24; 95% CI: 3.40, 8.08 for the default model). On the other hand, we found statistically significant positive associations with PTB and negative associations with BW. For PTB and first-trimester PM_10_, OR = 1.15 (95% CI: 0.87, 1.51) for 50–69 μg/m^3^ and OR = 1.45 (95% CI: 0.92, 2.26) for ≥ 70 μg/m^3^ compared with first-trimester PM_10_ < 30 μg/m^3^. Average birth weight was estimated to be 28 g lower (95% CI: –55.58, –1.21) for 50–69 μg/m^3^ during the first trimester. Other results were similar to those in Table 3. Tables S3–S5 report results when adjusting for one copollutant at a time. We found no major qualitative changes, although third-trimester associations with PM_10_ values ≥ 70 μg/m^3^ were higher when only adjusting for CO.

Table S6 shows the results of our third sensitivity check, which ran the analysis only for observations without missing values on eclampsia, hypertension, parity, and smoking. The results were similar to those in the core specification, suggesting that our treatment of missing observations did not compromise the findings.

Table S7 shows the results of our estimation when restricting the analysis to pregnancies conceived before or during the volcano eruptions. These were quite similar to those in the categorical core specification (Table 3), particularly for the associations with concentration levels ≥ 70 μg/m^3^. One difference with the core model was the statistically significant and negative association between exposure to PM_10_ during the first trimester and the odds of a PTB: OR = 0.65 (95% CI: 0.47, 0.90) for 30–49 μg/m^3^, OR = 0.61 (95% CI: 0.42, 0.88) for 50–69 μg/m^3^, and OR = 0.57 (95% CI: 0.34, 0.95) for ≥ 70 μg/m^3^ compared with first trimester PM_10_ < 30 μg/m^3^ in Table S7, versus OR = 0.92 (95% CI: 0.78, 1.08) for 30–49 μg/m^3^, OR = 0.85 (95% CI: 0.66, 1.08) for 50–69 μg/m^3^, and OR = 0.69 (95% CI: 0.47, 10.2) for ≥ 70 μg/m^3^ compared with first-trimester PM_10_ < 30 μg/m^3^ in Table 3.

In Table S8 we show results when restricting the analysis to pregnancies with a birth date after the first eruption. Again, the positive association between high levels of concentration of PM_10_ in the third trimester and PTB was robust to this change in the sample, although the OR was larger for PM_10_ ≥ 70 μg/m^3^ [OR = 14.27 (95% CI: 8.49, 23.98) in Table S8, versus OR = 5.24 (95% CI: 3.40, 8.08) in Table 3]. We also found some positive associations between high exposures to PM_10_ concentration in the first trimester and BW.

In Tables S9 and S10 we report associations from analyses that were restricted to pregnancies that were not exposed to the volcano eruptions. The associations between PTB and a 10-μg/m^3^ increase in PM_10_ during the third trimester were similar to the complete analysis, but not statistically significant (OR = 1.15; 95% CI: 0.93, 1.42 compared with OR = 1.10; 95% CI: 1.03, 1.19 based on the default model). We found, however, a positive association between PM_10_ levels between 30 and 49 μg/m^3^ and PTB (OR = 1.35; 95% CI: 1.03, 1.77) when estimating the categorical model.

Finally, the association between third-trimester PM_10_ and preterm persisted when we modeled residuals of trimester averages regressed on the average of a reference trimester to account for potential collinearity, as in Bell et al. (2007) (see Tables S11–S13).

## Discussion and Conclusions

We explored the effect of PM_10_ on PTB and on BW and LBW in full-term pregnancies. We took advantage of the fact that in 2011 the ashes and dust resulting from the eruption of the Puyehue volcano in Chile increased substantially the exposure to PM_10_ in Montevideo.

We found that high levels of PM_10_ concentration during the third trimester were positively associated with PTB in our study population. In particular, we estimated that a 10-μg/m^3^ increase in average of PM_10_ during the third trimester of pregnancy was associated with a 10% increase in the odds of a PTB (95% CI: 1.03, 1.19). Compared with third-trimester PM_10_ < 30 μg/m^3^, the odds of PTB in women with third-trimester PM_10_ ≥ 70 μg/m^3^ was about five times higher (OR = 5.24; 95% CI: 3.40, 8.08). These results were generally robust in terms of sign and statistical significance to alternate specifications that controlled for potentially confounding covariates and used different samples. They are also in line with prior results in the literature. For example, estimates from a meta-analysis conducted by Stieb et al. (2012) showed a pooled OR of the relationship between third-trimester PM_10_ and PTB of 1.06 (95% CI: 1.03, 1.11) per 20-μg/m^3^ increase of PM_10_. Also, Parker et al. (2008) found that a reduction in exposure to pollution due to the closure of a steel mill in Utah Valley decreased the likelihood of PTB. Although they attributed this finding to decreases in pollution in general, they did not explicitly quantify the relationship between PTB and specific pollutant levels. To our knowledge, ours is the first study using a natural experiment to report a positive and significant association between PM_10_ and PTB.

Unlike some prior studies (Dadvand et al. 2013; Parker et al. 2011; Rich et al. 2015; Šrám et al. 2005), we did not find adverse associations between PM_10_ and term BW or term LBW. On the contrary, results for some of our specifications suggest that exposures to high levels of PM_10_ in the second and third trimesters were associated with increases in BW and decreases in LBW for full-term births. Similar results have been reported by Stieb et al. (2012) and Edwards et al. (2015). Although these findings may appear counterintuitive, they could reflect selection effects. The association between higher exposures to PM_10_ during the second trimester and increases in BW (as well as decreases in LBW) could be the result of a higher risk of spontaneous abortions. Under this hypothesis, exposure to levels of PM_10_ ≥ 70 μg/m^3^ during the first weeks of the second trimester (before gestation week 20) would be associated with higher weight at birth only because the healthier babies survive the second trimester. Unfortunately, we cannot directly test this hypothesis due to lack of registries on aborted pregnancies in our data. However, recent literature has identified similar effects. In particular, there is evidence of statistical associations between ambient air pollutants and spontaneous abortions. Enkhmaa et al. (2014) correlated fetal deaths with mean monthly levels of various air pollutants by means of regression analysis. They used pollution data from Mongolia and 1,219 medical records of women who had a spontaneous abortion in the same country. The authors found a correlation of 80–90%, depending on the pollutant in consideration. Moridi et al. (2014) investigated the association between spontaneous abortion and ambient pollutants. The authors estimated the mean exposure to pollution for each of 296 women in Iran. They found odds ratios of abortion in the areas with higher concentrations of CO, NO_2_, ozone, and PM_10_ ranging between 0.94 and 1.98 (*p* < 0.05).

On the other hand, a potential explanation for the third-trimester results on BW and LBW is that they are selection artifacts derived from the negative effects of PM_10_ on PTB. If high levels of PM_10_ trigger preterm births that otherwise would not have occurred, and if these additional preterm births are also those with relative lower weight (i.e., affecting the most vulnerable babies), then BW should increase and LBW should decrease in pregnancies that reach full term. This hypothesis assumes that the selection effect stemming from higher levels of preterm births is sufficiently large to offset any negative effect of PM_10_ on intrauterine growth.

We believe this paper contributes to the literature on pollution and health in several ways. First, it is one of a few studies to investigate the association between pollution and perinatal health using a natural experiment. Our reliance on PM_10_ variation associated with the volcano eruption, together with the use of adjustors for individual-level characteristics, delivery hospital effects, and weather measures, provides internal validity to the study. In particular, our findings are less subject to the critique that results are driven by selection of poorer populations into polluted areas or determined by unobserved time trends correlated with pollutant trends. Nevertheless, we cannot rule out the possibility that exposure to PM from the volcano could have differed within the study area in relation to socioeconomic and other factors that might be associated with birth outcomes and that we could not control for.

Second, we study transitory and intense exposures to high levels of particulate matter in a city characterized by good air quality. Traditionally, Montevideo has registered 24-hr mean averages of PM_10_ that fall below WHO’s threshold of 50 μg/m^3^ (IMM 2008, 2009). Most other analyses deal with regions exposed to high levels of pollutants. Our results are consistent with the hypothesis that even short and acute exposures have effects on health at birth.

Third, our categorical analysis identifies specific ranges for which PM_10_ can have particularly severe consequences on public health. It contributes, in this way, to the formulation of concrete recommendations for public action in the management of ambient air emergencies. This includes, for example, issuing notices recommending that pregnant women stay inside during such episodes.

Finally, we provide new evidence of the association between PM_10_ and perinatal health in a developing country, and particularly in Latin America, where the evidence is scarce (Edwards et al. 2015). This is important because underlying conditions may differ according to the country’s level of development, and the effects of pollution may be heterogeneous in these features. In our case, one of such differing conditions may be the maternal education level: More than 30% of the mothers in our sample did not finish middle school. Another condition may be the quality of health services.

Our analysis would be biased if the volcano eruptions were spuriously correlated with changes in the composition of pregnant women over time. Ideally, comparing outcomes for the same mother across her different pregnancies would avoid this problem. Unfortunately we were unable to identify multiple pregnancies by the same mother in our data.

To sum up, our results suggest that exposure to high levels of PM_10_ during the third trimester increased PTB among residents of Montevideo, a city with episodes of high air pollution levels resulting from eruptions of the Puyehue volcano. However, we did not find associations between these exposures and BW or LBW among full-term pregnancies. Future research should gain insight on the physiological mechanisms behind these associations.

***Editor’s Note:** In the Advance Publication, the Results section of the Abstract stated “Exposures were not consistently associated with birth weight or preterm birth among term births, though second trimester exposures were associated with higher birth weight, contrary to expectations.” The correct sentence is “Exposures were not consistently associated with birth weight or low birth weight among term births, though second trimester exposures were associated with higher birth weight, contrary to expectations.”*

*The correction has been made in the final published article. *EHP* and the authors regret the error.*

## Supplemental Material

(294 KB) PDF
