# Supplementary material for: Exposures to Particulate Matter from the Eruptions of the Puyehue Volcano and Birth Outcomes in Montevideo, Uruguay
Source: Environ Health Perspect. 2016 May 6;124(11):1816–22. doi: 10.1289/EHP235 (PMC5089889; doi:10.1289/EHP235)
Supplement: (294 KB) PDF [file EHP235.s001.acco.pdf]

Note to readers with disabilities: *EHP* strives to ensure that all journal content is accessible to all readers. However, some figures and Supplemental Material published in *EHP* articles may not conform to 508 standards due to the complexity of the information being presented. If you need assistance accessing journal content, please contact ehp508@niehs.nih.gov. Our staff will work with you to assess and meet your accessibility needs within 3 working days.

Supplemental Material

Exposures to Particulate Matter from the Eruptions of the Puyehue Volcano and Birth Outcomes in Montevideo, Uruguay

Ana Ines Balsa, Marcelo Caffera, and Juanita Bloomfield

Table of Contents

Table S1: categorical analysis controlling for activity in thermal power plants and oil refinery (all pregnancies, n=79,328; term births, n=72,920)

Table S2: Categorical effects of PM10 on birth outcomes controlling for CO, NO_2, SO_2 (all pregnancies, n=79,252; term births, n=72,920)

Table S3: Categorical effects of PM10 on birth outcomes controlling for CO (all pregnancies, n=79,297; term births, n=72,920)

Table S4: Categorical effects of PM10 on birth outcomes controlling for SO_2 (all pregnancies, N=79,323; term births, N=72,920)

Table S5: Categorical effects of PM10 on birth outcomes controlling for NO_2 (all pregnancies, n=79,277; term births, n=72,920)

Table S6: Excluding observations with missing values on eclampsia, hypertension, parity, and smoking (all pregnancies, n=63,865; term births, n=58,713)

Table S7: Categorical effects of PM10 on birth outcomes for pregnancies conceived before or during the volcano eruptions (all pregnancies, n=51,106; term births, n=46,883)

Table S8: Categorical effects of PM10 on birth outcomes for pregnancies conceived during or after the volcano eruptions (all pregnancies, n=53,969; term births, n=50,147 (BW), n=50,135 (LBW))

Table S9: Effects of average PM10 concentration on birth outcomes for pregnancies not exposed to the volcano eruptions (all pregnancies, n=53,062; term births, n=48,699)
Table S10: Categorical effects of PM10 on birth outcomes for pregnancies not exposed to the volcano eruptions (all pregnancies, n=53,019; term births, n=48,699)
Table S11: Substituting 1st trimester pollution and weather values for residuals of these measures on their respective values for the 2nd and 3rd trimesters (all pregnancies, n=79,328; term births, n=72,920)
Table S12: Substituting 2nd trimester pollution and weather values for residuals of these measures on their respective values for 1st and 3rd trimesters (all pregnancies, n=79,328; term births, n=72,920)
Table S13: Substituting 3rd trimester pollution and weather value0s for residuals of these measures on their respective values for the 1st and 2nd trimesters (all pregnancies, n=79,328; term births, n=72,920)

Table S1: categorical analysis controlling for activity in thermal power plants and oil refinery (all pregnancies, n=79,328; term births, n=72,920)

	All pregnancies	Only full term pregnancies	
	Preterm Birth	Birth weight	LBW
Characteristic	(1)	(2)	(3)
$30 \leq PM_{10} \leq 49$ 1st trimester	0.98 [0.82,1.17]	-11.43 [-31.15,8.29]	1.04 [0.78,1.38]
$50 \leq PM_{10} \leq 69$ 1st trimester	0.81 [0.62,1.06]	-3.67 [-33.03,25.69]	0.98 [0.63,1.51]
$70 \leq PM_{10}$ 1st trimester	0.58 [0.36,0.92]*	-29.13 [-82.08,23.82]	1.24 [0.59,2.60]
$30 \leq PM_{10} \leq 49$ 2nd trimester	0.80 [0.64,0.99]*	16.97 [-4.14,38.08]	1.11 [0.82,1.50]
$50 \leq PM_{10} \leq 69$ 2nd trimester	0.67 [0.49,0.91]**	32.76 [-0.17,65.69]#	1.22 [0.76,1.96]
$70 \leq PM_{10}$ 2nd trimester	0.69 [0.44,1.11]	118.09 [69.43,166.76]**	0.82 [0.40,1.68]
$30 \leq PM_{10} \leq 49$ 3rd trimester	0.93 [0.77,1.11]	-2.29 [-21.78,17.21]	0.79 [0.60,1.04]#
$50 \leq PM_{10} \leq 69$ 3rd trimester	1.34 [0.98,1.84]#	68.24 [37.50,98.99]**	0.49 [0.32,0.76]**
$70 \leq PM_{10}$ 3rd trimester	4.99 [3.11,8.00]**	16.83 [-26.70,60.37]	0.74 [0.40,1.36]

Notes: Odd ratios for dichotomous variables (PTB, LBW), coefficients for continuous variables (BW). Confidence intervals in parentheses. Apart from the variables mentioned in the title of the table, the regressions adjust for maternal age, education, and marital status, pregnancy conditions, maternal smoking status, onset of prenatal care, temperature, rain, humidity, air pressure, and wind intensity in each trimester of pregnancy, indicators for calendar quarter of gestation (a dichotomous indicator for each calendar quarter between June 2009 and March 2013), and indicators for prenatal care center. Both for the PM10 and weather variables, 3rd trimester values are averaged across gestation weeks 28 and 36 (or an earlier week if the pregnancy did not reach full term) when the outcome is PTB. In the case of BW or LBW, 3rd trimester averages consider the full length of the trimester until birth. **p<0.01; *p<0.05; # p<0.1.

Table S2: Categorical effects of PM10 on birth outcomes controlling for CO, NO_2, SO_2 (all pregnancies, n=79,252; term births, n=72,920)

	All pregnancies	Only full term pregnancies	
	Prematurity	Birth weight	LBW
Characteristic	(1)	(2)	(3)
$30 \leq PM_{10} \leq 49$ 1st trimester	1.09<br>[0.93,1.29]	-30.77<br>[-49.54,-11.99]**	1.16<br>[0.889,1.515]
$50 \leq PM_{10} \leq 69$ 1st trimester	1.15<br>[0.87,1.51]	-28.4<br>[-55.58,-1.21]*	1.15<br>[0.771,1.716]
$70 \leq PM_{10}$ 1st trimester	1.45<br>[0.92,2.26]	-25.89<br>[-78.46,26.68]	1.28<br>[0.612,2.688]
$30 \leq PM_{10} \leq 49$ 2nd trimester	0.92<br>[0.74,1.13]	15.06<br>[-5.53,35.65]	0.98<br>[0.728,1.316]
$50 \leq PM_{10} \leq 69$ 2nd trimester	1.26<br>[0.94,1.68]	51.67<br>[17.74,85.60]**	1<br>[0.625,1.597]
$70 \leq PM_{10}$ 2nd trimester	1.02<br>[0.67,1.56]	123.22<br>[76.40,170.03]**	0.7<br>[0.360,1.377]
$30 \leq PM_{10} \leq 49$ 3rd trimester	0.98<br>[0.83,1.17]	-8.06<br>[-26.84,10.72]	0.86<br>[0.658,1.137]
$50 \leq PM_{10} \leq 69$ 3rd trimester	1.67<br>[1.19,2.35]**	89.51<br>[58.00,121.02]**	0.43<br>[0.277,0.661]**
$70 \leq PM_{10}$ 3rd trimester	16.35<br>[9.26,28.88]**	66.84<br>[19.29,114.39]**	0.52<br>[0.275,0.997]*

.

Notes: Odd ratios for dichotomous variables (PTB, LBW), coefficients for continuous variables (BW). Confidence intervals in parentheses. Apart from the variables mentioned in the title of the table, the regressions adjust for maternal age, education, and marital status, pregnancy conditions, maternal smoking status, onset of prenatal care, temperature, rain, humidity, air pressure, and wind intensity in each trimester of pregnancy, indicators for calendar quarter of gestation (a dichotomous indicator for each calendar quarter between June 2009 and March 2013), and indicators for prenatal care center. Both for the PM10 and weather variables, 3rd trimester values are averaged across gestation weeks 28 and 36 (or an earlier week if the pregnancy did not reach full term) when the outcome is PTB. In the case of BW or LBW, 3rd trimester averages consider the full length of the trimester until birth. **p<0.01; *p<0.05.

Table S3: Categorical effects of PM10 on birth outcomes controlling for CO (all pregnancies, n=79,297; term births, n=72,920)

	All pregnancies	Only full term pregnancies	
	Preterm Birth	Birth weight	LBW
Characteristic	(1)	(2)	(3)
$30 \leq PM_{10} \leq 49$ 1st trimester	1.01 [0.85,1.18]	-18.12 [-35.41,-0.83]*	1.1 [0.865,1.408]
$50 \leq PM_{10} \leq 69$ 1st trimester	1.11 [0.86,1.43]	-10.13 [-35.40,15.14]	1.04 [0.719,1.507]
$70 \leq PM_{10}$ 1st trimester	1.58 [1.02,2.45]*	-18.53 [-68.75,31.68]	1.26 [0.629,2.522]
$30 \leq PM_{10} \leq 49$ 2nd trimester	0.79 [0.64,0.97]*	20.72 [1.08,40.35]*	0.97 [0.731,1.280]
$50 \leq PM_{10} \leq 69$ 2nd trimester	1.14 [0.87,1.51]	49.49 [17.25,81.73]**	1.05 [0.674,1.652]
$70 \leq PM_{10}$ 2nd trimester	1.07 [0.70,1.62]	124.69 [79.99,169.39]**	0.71 [0.377,1.352]
$30 \leq PM_{10} \leq 49$ 3rd trimester	0.97 [0.82,1.15]	-4.84 [-23.19,13.52]	0.82 [0.627,1.076]
$50 \leq PM_{10} \leq 69$ 3rd trimester	2.03 [1.48,2.78]**	90.44 [59.51,121.36]**	0.43 [0.284,0.666]**
$70 \leq PM_{10}$ 3rd trimester	18.76 [10.81,32.54]**	58.46 [12.26,104.66]*	0.6 [0.318,1.115]

Notes: Odd ratios for dichotomous variables (PTB, LBW), coefficients for continuous variables (BW). Confidence intervals in parentheses. Apart from the variable mentioned in the title of the table, the regressions adjust for maternal age, education, and marital status, pregnancy conditions, maternal smoking status, onset of prenatal care, temperature, rain, humidity, air pressure, and wind intensity in each trimester of pregnancy, indicators for calendar quarter of gestation (a dichotomous indicator for each calendar quarter between June 2009 and March 2013), and indicators for prenatal care center. Both for the PM10 and weather variables, 3rd trimester values are averaged across gestation weeks 28 and 36 (or an earlier week if the pregnancy did not reach full term) when the outcome is PTB. In the case of BW or LBW, 3rd trimester averages consider the full length of the trimester until birth. **p<0.01; *p<0.05; # p<0.1.

Table S4: Categorical effects of PM10 on birth outcomes controlling for SO_2 (all pregnancies, N=79,323; term births, N=72,920)

	All pregnancies	Only full term pregnancies	
	Preterm Birth	Birth weight	LBW
Characteristic	(1)	(2)	(3)
$30 \leq PM_{10} \leq 49$ 1st trimester	1.05 [0.89,1.25]	-20.06 [-38.07,-2.05]*	1.08 [0.834,1.397]
$50 \leq PM_{10} \leq 69$ 1st trimester	1.12 [0.85,1.46]	-30.36 [-57.22,-3.50]*	1.12 [0.755,1.662]
$70 \leq PM_{10}$ 1st trimester	1.03 [0.68,1.56]	-56.43 [-105.52,-7.34]*	1.44 [0.718,2.881]
$30 \leq PM_{10} \leq 49$ 2nd trimester	0.88 [0.71,1.09]	11.34 [-8.73,31.42]	0.99 [0.744,1.313]
$50 \leq PM_{10} \leq 69$ 2nd trimester	1.01 [0.77,1.33]	27.26 [-4.04,58.57]#	1.11 [0.709,1.727]
$70 \leq PM_{10}$ 2nd trimester	1.14 [0.76,1.71]	93.22 [49.11,137.34]**	0.8 [0.423,1.512]
$30 \leq PM_{10} \leq 49$ 3rd trimester	1.01 [0.86,1.19]	-1.68 [-19.91,16.55]	0.8 [0.614,1.044]
$50 \leq PM_{10} \leq 69$ 3rd trimester	1.4 [1.02,1.90]*	72.82 [43.43,102.21]**	0.48 [0.318,0.718]**
$70 \leq PM_{10}$ 3rd trimester	6.61 [4.29,10.20]**	30.91 [-12.34,74.16]	0.7 [0.382,1.267]

Notes: Odd ratios for dichotomous variables (PTB, LBW), coefficients for continuous variables (BW). Confidence intervals in parentheses. Apart from the variable mentioned in the title of the table, the regressions adjust for maternal age, education, and marital status, pregnancy conditions, maternal smoking status, onset of prenatal care, temperature, rain, humidity, air pressure, and wind intensity in each trimester of pregnancy, indicators for calendar quarter of gestation (a dichotomous indicator for each calendar quarter between June 2009 and March 2013), and indicators for prenatal care center. Both for the PM10 and weather variables, 3rd trimester values are averaged across gestation weeks 28 and 36 (or an earlier week if the pregnancy did not reach full term) when the outcome is PTB. In the case of BW or LBW, 3rd trimester averages consider the full length of the trimester until birth. **p<0.01; *p<0.05; # p<0.1.

Table S5: Categorical effects of PM10 on birth outcomes controlling for NO_2 (all pregnancies, n=79,277; term births, n=72,920)

Characteristic	All pregnancies<br>Preterm birth<br>(1)	Only full term pregnancies<br>Birth weight<br>(2)	 LBW<br>(3)
$30 \le PM_{10} \le 49$ 1st trimester	0.94<br>[0.80,1.10]	[-33.01,1.16]#<br>-7.63	1.1<br>[0.869,1.398]
$50 \le PM_{10} \le 69$ 1st trimester	0.79<br>[0.61,1.02]#	[-32.38,17.11]<br>-10.58	0.97<br>[0.675,1.385]
$70 \le PM_{10}$ 1st trimester	0.59<br>[0.40,0.87]**	[-57.65,36.50]<br>19.22	1.04<br>[0.538,2.029]
$30 \le PM_{10} \le 49$ 2nd trimester	0.83<br>[0.67,1.03]#	[-0.23,38.67]#<br>45.41	0.98<br>[0.741,1.290]
$50 \le PM_{10} \le 69$ 2nd trimester	0.76<br>[0.59,0.99]*	[14.78,76.03]**<br>118.09	1.05<br>[0.675,1.623]
$70 \le PM_{10}$ 2nd trimester	0.75<br>[0.51,1.12]	[73.95,162.23]**<br>-14.13	0.7<br>[0.368,1.338]
$30 \le PM_{10} \le 49$ 3rd trimester	1<br>[0.85,1.18]	[-32.42,4.16]<br>54.11	0.87<br>[0.669,1.143]
$50 \le PM_{10} \le 69$ 3rd trimester	1.29<br>[0.96,1.74]#	[25.18,83.04]**<br>29.23	0.54<br>[0.364,0.812]**
$70 \le PM_{10}$ 3rd trimester	5.01<br>[3.19,7.87]**	[-14.87,73.33]<br>12.89	0.73<br>[0.396,1.343]

Notes: Odd ratios for dichotomous variables (PTB, LBW), coefficients for continuous variables (BW). Confidence intervals in parentheses. Apart from the variable mentioned in the title of the table, the regressions adjust for maternal age, education, and marital status, pregnancy conditions, maternal smoking status, onset of prenatal care, temperature, rain, humidity, air pressure, and wind intensity in each trimester of pregnancy, indicators for calendar quarter of gestation (a dichotomous indicator for each calendar quarter between June 2009 and March 2013), and indicators for prenatal care center. Both for the PM10 and weather variables, 3rd trimester values are averaged across gestation weeks 28 and 36 (or an earlier week if the pregnancy did not reach full term) when the outcome is PTB. In the case of BW or LBW, 3rd trimester averages consider the full length of the trimester until birth. **p<0.01; *p<0.05; # p<0.1.

Table S6: Excluding observations with missing values on eclampsia, hypertension, parity, and smoking (all pregnancies, n=63,865; term births, n=58,713)

	All pregnancies	Only full term pregnancies	
	Preterm Birth	Birth weight	LBW
Characteristic	(1)	(2)	(3)
$30 \leq PM_{10} \leq 49$ 1st trimester	0.90<br>[0.75,1.08]	-12.56<br>[-31.09,5.97]	1.04<br>[0.80,1.36]
$50 \leq PM_{10} \leq 69$ 1st trimester	0.79<br>[0.60,1.04]#	-10.41<br>[-37.65,16.83]	1.02<br>[0.69,1.52]
$70 \leq PM_{10}$ 1st trimester	0.59<br>[0.38,0.91]*	-30.01<br>[-82.20,22.19]	1.44<br>[0.70,2.96]
$30 \leq PM_{10} \leq 49$ 2nd trimester	0.77<br>[0.61,0.97]*	11.06<br>[-10.50,32.62]	0.93<br>[0.69,1.26]
$50 \leq PM_{10} \leq 69$ 2nd trimester	0.77<br>[0.58,1.02]#	28.72<br>[-4.27,61.71]#	1.20<br>[0.75,1.91]
$70 \leq PM_{10}$ 2nd trimester	0.86<br>[0.56,1.33]	103.46<br>[55.39,151.53]**	0.76<br>[0.38,1.53]
$30 \leq PM_{10} \leq 49$ 3rd trimester	0.7<br>[0.81,1.16]	-14.78<br>[-35.02,5.45]	0.83<br>[0.62,1.12]
$50 \leq PM_{10} \leq 69$ 3rd trimester	1.36<br>[0.98,1.87]#	48.99<br>[17.31,80.67]**	0.49<br>[0.32,0.77]**
$70 \leq PM_{10}$ 3rd trimester	6.04<br>[3.75,9.75]**	30.88<br>[-16.61,78.37]	0.71<br>[0.36,1.37]

Notes: Odd ratios for dichotomous variables (PTB, LBW), coefficients for continuous variables (BW). Confidence intervals in parentheses. Regression adjusts for maternal age, education, and marital status, pregnancy conditions, maternal smoking status, onset of prenatal care, temperature, rain, humidity, air pressure, and wind intensity in each trimester of pregnancy, indicators for calendar quarter of gestation (a dichotomous indicator for each calendar quarter between June 2009 and March 2013), and indicators for prenatal care center. Both for the PM10 and weather variables, 3rd trimester values are averaged across gestation weeks 28 and 36 (or an earlier week if the pregnancy did not reach full term) when the outcome is PTB. In the case of BW or LBW, 3rd trimester averages consider the full length of the trimester until birth. **p<0.01; *p<0.05; # p<0.1.

Table S7: Categorical effects of PM10 on birth outcomes for pregnancies conceived before or during the volcano eruptions (all pregnancies, n=51,106; term births, n=46,883)

	All pregnancies	Only full term pregnancies	
	Preterm Birth	Birth weight	LBW
Characteristic	(1)	(2)	(3)
$30 \leq PM_{10} \leq 49$ 1st trimester	0.65 [0.47,0.90]**	16.56 [-17.95,51.06]	1.12 [0.68,1.85]
$50 \leq PM_{10} \leq 69$ 1st trimester	0.61 [0.42,0.88]**	14.08 [-25.94,54.10]	1.11 [0.61,1.99]
$70 \leq PM_{10}$ 1st trimester	0.57 [0.34,0.95]*	-13.92 [-71.47,43.63]	1.38 [0.61,3.13]
$30 \leq PM_{10} \leq 49$ 2nd trimester	0.51 [0.36,0.71]**	32.65 [-0.16,65.46]#	0.62 [0.37,1.01]#
$50 \leq PM_{10} \leq 69$ 2nd trimester	0.46 [0.32,0.66]**	43.06 [4.50,81.62]*	0.71 [0.39,1.29]
$70 \leq PM_{10}$ 2nd trimester	0.51 [0.33,0.81]**	130.61 [80.88,180.34]**	0.46 [0.22,0.98]*
$30 \leq PM_{10} \leq 49$ 3rd trimester	1.08 [0.88,1.33]	-0.32 [-20.78,20.14]	0.75 [0.56,1.00]#
$50 \leq PM_{10} \leq 69$ 3rd trimester	1.65 [1.20,2.27]**	84.70 [51.76,117.64]**	0.48 [0.30,0.76]**
$70 \leq PM_{10}$ 3rd trimester	7.03 [4.25,11.62]**	50.45 [1.10,99.80]*	0.76 [0.38,1.53]

Notes: Odd ratios for dichotomous variables (PTB, LBW), coefficients for continuous variables (BW). Confidence intervals in parentheses. Regression adjusts for maternal age, education, and marital status, pregnancy conditions, maternal smoking status, onset of prenatal care, temperature, rain, humidity, air pressure, and wind intensity in each trimester of pregnancy, indicators for calendar quarter of gestation (a dichotomous indicator for each calendar quarter between June 2009 and March 2013), and indicators for prenatal care center. Both for the PM10 and weather variables, 3rd trimester values are averaged across gestation weeks 28 and 36 (or an earlier week if the pregnancy did not reach full term) when the outcome is PTB. In the case of BW or LBW, 3rd trimester averages consider the full length of the trimester until birth. **p<0.01; *p<0.05; # p<0.1

Table S8: Categorical effects of PM10 on birth outcomes for pregnancies conceived during or after the volcano eruptions (all pregnancies, n=53,969; term births, n=50,147 (BW), n=50,135 (LBW))

	All pregnancies	Only full term pregnancies	
	Preterm Birth	Birth weight	LBW
Characteristic	(1)	(2)	(3)
$30 \leq PM_{10} \leq 49$ 1st trimester	1.08<br>[0.89,1.32]	-25.48<br>[-45.88,-5.08]*	1.23<br>[0.90,1.68]
$50 \leq PM_{10} \leq 69$ 1st trimester	0.96<br>[0.69,1.31]	-21.71<br>[-50.36,6.93]	1.17<br>[0.77,1.79]
$70 \leq PM_{10}$ 1st trimester	0.70<br>[0.44,1.13]	-50.14<br>[-102.41,2.12]#	1.65<br>[0.77,3.53]
$30 \leq PM_{10} \leq 49$ 2nd trimester	0.95<br>[0.75,1.21]	3.23<br>[-17.59,24.05]	1.14<br>[0.84,1.53]
$50 \leq PM_{10} \leq 69$ 2nd trimester	1.06<br>[0.77,1.47]	23.11<br>[-11.75,57.96]	1.37<br>[0.81,2.30]
$70 \leq PM_{10}$ 2nd trimester	1.49<br>[0.94,2.37]#	96.91<br>[47.80,146.01]**	0.99<br>[0.47,2.06]
$30 \leq PM_{10} \leq 49$ 3rd trimester	0.85<br>[0.68,1.06]	13.63<br>[-9.63,36.89]	0.79<br>[0.57,1.10]
$50 \leq PM_{10} \leq 69$ 3rd trimester	1.55<br>[1.09,2.20]*	69.31<br>[35.54,103.09]**	0.56<br>[0.34,0.93]*
$70 \leq PM_{10}$ 3rd trimester	14.27<br>[8.49,23.98]**	27.04<br>[-20.95,75.03]	0.92<br>[0.46,1.85]

Notes: Odd ratios for dichotomous variables (PTB, LBW), coefficients for continuous variables (BW). Confidence intervals in parentheses. Regression adjusts for maternal age, education, and marital status, pregnancy conditions, maternal smoking status, onset of prenatal care, temperature, rain, humidity, air pressure, and wind intensity in each trimester of pregnancy, indicators for calendar quarter of gestation (a dichotomous indicator for each calendar quarter between June 2009 and March 2013), and indicators for prenatal care center. Both for the PM10 and weather variables, 3rd trimester values are averaged across gestation weeks 28 and 36 (or an earlier week if the pregnancy did not reach full term) when the outcome is PTB. In the case of BW or LBW, 3rd trimester averages consider the full length of the trimester until birth. **p<0.01; *p<0.05; # p<0.1.

Table S9: Effects of average PM10 concentration on birth outcomes for pregnancies not exposed to the volcano eruptions (all pregnancies, n=53,062; term births, n=48,699)

	All pregnancies	Only full term pregnancies	
	Preterm Birth	Birth weight	LBW
Characteristic	(1)	(2)	(3)
PM10 1st trimester (per 10 µg/m3 increase)	1.13<br>[0.99,1.29]#	-10.92<br>[-25.50,3.65]	1.04<br>[0.86,1.27]
PM10 2nd trimester (per 10 µg/m3 increase)	1.06<br>[0.85,1.33]	4.09<br>[-23.29,31.47]	1.00<br>[0.69,1.46]
PM10 3rd trimester (per 10 µg/m3 increase)	1.15<br>[0.93,1.42]	24.44<br>[0.88,48.00]*	0.77<br>[0.56,1.07]

Notes: Odd ratios for dichotomous variables (PTB, LBW), coefficients for continuous variables (BW). Confidence intervals in parentheses. Regression adjusts for maternal age, education, and marital status, pregnancy conditions, maternal smoking status, onset of prenatal care, temperature, rain, humidity, air pressure, and wind intensity in each trimester of pregnancy, indicators for calendar quarter of gestation (a dichotomous indicator for each calendar quarter between June 2009 and March 2013), and indicators for prenatal care center. Both for the PM10 and weather variables, 3rd trimester values are averaged across gestation weeks 28 and 36 (or an earlier week if the pregnancy did not reach full term) when the outcome is PTB. In the case of BW or LBW, 3rd trimester averages consider the full length of the trimester until birth. *p<0.05; # p<0.1. Sample includes pregnancies with a date of delivery before June 8th 2011 and pregnancies with a date of conception after December 30th 2011.

Table S10: Categorical effects of PM10 on birth outcomes for pregnancies not exposed to the volcano eruptions (all pregnancies, n=53,019; term births, n=48,699)

	All pregnancies	Only full term pregnancies	
	Preterm Birth	Birth weight	LBW
Characteristic	(1)	(2)	(3)
$30 \leq PM_{10} \leq 49$ 1st trimester	1.17 [0.98,1.39]#	-27.45 [-46.47,-8.43]**	1.11 [0.86,1.43]
$30 \leq PM_{10} \leq 49$ 2nd trimester	1.17 [0.86,1.60]	16.42 [-13.18,46.03]	1.15 [0.77,1.72]
$30 \leq PM_{10} \leq 49$ 3rd trimester	1.35 [1.03,1.77]*	-21.31 [-57.26,14.65]	0.81 [0.47,1.41]
$50 \leq PM_{10} \leq 69$ 3rd trimester	N/A	43.42 [-47.01,133.84]	0.47 [0.14,1.57]

Notes: Odd ratios for dichotomous variables (PTB, LBW), coefficients for continuous variables (BW). Confidence intervals in parentheses. Regression adjusts for maternal age, education, and marital status, pregnancy conditions, maternal smoking status, onset of prenatal care, temperature, rain, humidity, air pressure, and wind intensity in each trimester of pregnancy, indicators for calendar quarter of gestation (a dichotomous indicator for each calendar quarter between June 2009 and March 2013), and indicators for prenatal care center. Both for the PM10 and weather variables, 3rd trimester values are averaged across gestation weeks 28 and 36 (or an earlier week if the pregnancy did not reach full term) when the outcome is PTB. In the case of BW or LBW, 3rd trimester averages consider the full length of the trimester until birth. **p<0.01; *p<0.05; # p<0.1. Sample includes pregnancies with a date of delivery before June 8th 2011 and pregnancies with a date of conception after December 30th 2011. N/A – not applicable. Only 156 observations (out of 53062) were exposed to trimester average levels of PM10 above 50 µg/m3 in the period.

Table S11: Substituting 1st trimester pollution and weather values for residuals of these measures on their respective values for the 2nd and 3rd trimesters (all pregnancies, n=79,328; term births, n=72,920)

	All pregnancies	Only full term pregnancies	
	Preterm Birth	Birth weight	LBW
Characteristic	(1)	(2)	(3)
PM10 1st trimester (per 10 µg/m3 increase)	0.99	-3.03	1.04
	[0.91,1.06]	[-11.27,5.22]	[0.93,1.17]
PM10 2nd trimester (per 10 µg/m3 increase)	0.95	10.84	1.04
	[0.86,1.06]	[0.12,21.57]*	[0.89,1.22]
PM10 3rd trimester (per 10 µg/m3 increase)	1.11	-5.39	0.93
	[1.03,1.19]**	[-14.73,3.95]	[0.80,1.08]

Notes: Odd ratios for dichotomous variables (PTB, LBW), coefficients for continuous variables (BW). Confidence intervals in parentheses. Regression adjusts for maternal age, education, and marital status, pregnancy conditions, maternal smoking status, onset of prenatal care, temperature, rain, humidity, air pressure, and wind intensity in each trimester of pregnancy, indicators for calendar quarter of gestation (a dichotomous indicator for each calendar quarter between June 2009 and March 2013), and indicators for prenatal care center. Both for the PM10 and weather variables, 3rd trimester values are averaged across gestation weeks 28 and 36 (or an earlier week if the pregnancy did not reach full term) when the outcome is PTB. In the case of BW or LBW, 3rd trimester averages consider the full length of the trimester until birth. **p<0.01; *p<0.05; # p<0.1.

Table S12: Substituting 2nd trimester pollution and weather values for residuals of these measures on their respective values for 1st and 3rd trimesters (all pregnancies, n=79,328; term births, n=72,920)

	All pregnancies	Only full term pregnancies	
	Preterm Birth	Birth weight	LBW
Characteristic	(1)	(2)	(3)
PM10 1st trimester (per 10 µg/m3 increase)	0.97	3.00	1.050
	[0.89,1.06]	[-6.09,12.10]	[0.92,1.20]
PM10 2nd trimester (per 10 µg/m3 increase)	0.96	13.10	1.01
	[0.89,1.05]	[4.07,22.13]**	[0.89,1.15]
PM10 3rd trimester (per 10 µg/m3 increase)	1.09	0.40	0.94
	[1.00,1.18]*	[-9.83,10.62]	[0.81,1.10]

Notes: Odd ratios for dichotomous variables (PTB, LBW), coefficients for continuous variables (BW). Confidence intervals in parentheses. Regression adjusts for maternal age, education, and marital status, pregnancy conditions, maternal smoking status, onset of prenatal care, temperature, rain, humidity, air pressure, and wind intensity in each trimester of pregnancy, indicators for calendar quarter of gestation (a dichotomous indicator for each calendar quarter between June 2009 and March 2013), and indicators for prenatal care center. Both for the PM10 and weather variables, 3rd trimester values are averaged across gestation weeks 28 and 36 (or an earlier week if the pregnancy did not reach full term) when the outcome is PTB. In the case of BW or LBW, 3rd trimester averages consider the full length of the trimester until birth. **p<0.01; *p<0.05.

Table S13: Substituting 3rd trimester pollution and weather value0s for residuals of these measures on their respective values for the 1st and 2nd trimesters (all pregnancies, n=79,328; term births, n=72,920)

	All pregnancies	Only full term pregnancies	
	Preterm Birth	Birth weight	LBW
Characteristic	(1)	(2)	(3)
PM10 1st trimester (per 10 μg/m3 increase)	0.98<br>[0.90,1.056]	-2.31<br>[-10.80,6.19]	1.05<br>[0.93,1.19]
PM10 2nd trimester (per 10 μg/m3 increase)	1.04<br>[0.94,1.15]	8.77<br>[-2.78,20.32]	0.96<br>[0.81,1.14]
PM10 3rd trimester (per 10 μg/m3 increase)	1.10<br>[1.03,1.19]**	-5.78<br>[-14.90,3.35]	0.94<br>[0.81,1.08]

Notes: Odd ratios for dichotomous variables (PTB, LBW), coefficients for continuous variables (BW). Confidence intervals in parentheses. Regression adjusts for maternal age, education, and marital status, pregnancy conditions, maternal smoking status, onset of prenatal care, temperature, rain, humidity, air pressure, and wind intensity in each trimester of pregnancy, indicators for calendar quarter of gestation (a dichotomous indicator for each calendar quarter between June 2009 and March 2013), and indicators for prenatal care center. Both for the PM10 and weather variables, 3rd trimester values are averaged across gestation weeks 28 and 36 (or an earlier week if the pregnancy did not reach full term) when the outcome is PTB. In the case of BW or LBW, 3rd trimester averages consider the full length of the trimester until birth. **p<0.01; *p<0.05;.
